# Marginal and internal fit evaluation of conventional metal-ceramic versus zirconia CAD/CAM crowns

**DOI:** 10.4317/jced.55946

**Published:** 2020-01-01

**Authors:** Nayana Paul, K. N Raghavendra Swamy, M. R Dhakshaini, Sanjukta Sowmya, M. B Ravi

**Affiliations:** 1 PG Student, Department of Prosthodontics and Crown & Bridge, J.S.S. Dental College & Hospital, a Constituent College of JSS Academy of Higher Education and Research, Mysuru, Karnataka, India; 2 Professor and head, Department of Prosthodontics and Crown & Bridge, J.S.S. Dental College & Hospital, a Constituent College of JSS Academy of Higher Education and Research, Mysuru, Karnataka, India; 3 Professor, Department of Prosthodontics and Crown & Bridge, J.S.S. Dental College & Hospital, a Constituent College of JSS Academy of Higher Education and Research, Mysuru, Karnataka, India; 4 Lecturer, JSS Dental College and Hospital, a Constituent College of JSS Academy of Higher Education and Research, Mysuru, Karnataka, India; 5 Reader, JSS Dental College and Hospital, a Constituent College of JSS Academy of Higher Education and Research, Mysuru, Karnataka, India

## Abstract

**Background:**

The purpose of this *in vivo* study was to compare the marginal and internal gap widths of monolithic zirconia crowns fabricated by CAD/CAM technique and metal-ceramic crowns fabricated by conventional technique.

**Material and Methods:**

10 participants needing a single restoration were selected. Zirconia crowns using CAD/CAM technology (Group A) (n=10) and metal-ceramic crowns (Group B) (n=10) using lost wax casting technique were fabricated for each selected tooth. The marginal and internal gaps of crowns were recorded using a replica technique with light body silicone material stabilized with a regular set putty. Each replica was sectioned buccolingually and mesiodistally and then evaluated at five pre-determined sites. The points measured were PM for marginal gap, PA for axial gap, PAO for axio-occlusal transition gap and PO and PCO for occlusal gaps using a stereomicroscope at 30× magniﬁcation. The Paired Sample (t) test was used to detect significant differences between the two groups in terms of marginal and internal fit (α= 0.05).

**Results:**

The mean for the marginal gap was 77.42μm (±39.5μm) for Group A compared with 95.86μm (±55.12μm) for Group B. Mean values for internal gap was 87.24 (±21.7 µm) for Group A and 132.91 µm (± 50.63 µm) for Group B. Significant differences were observed between both the groups for marginal (*p*=.010) and internal (*p*=.000) fit.

**Conclusions:**

The CAD/CAM fabricated zirconia crowns demonstrated a better accuracy of fit when compared to metal-ceramic crowns fabricated by conventional technology.

** Key words:**Marginal fit, Internal fit, Computer-aided design/computer-aided manufacturing (CAD/CAM).

## Introduction

Metal-ceramic crowns remain the most commonly used crowns for fabricating full coverage restorations, since they combine the high strength properties of metal with the cosmetic appearance of ceramic (1). The conventional technique for fabricating a metal substructure is the lost wax technique and the use of various alloys for casting. However, coming of age all-ceramic restorations are gaining a lot of popularity. A number of different types of ceramic systems are now available for clinical use, lithium disilicate and zirconia being the most common.

The development of computer-aided design (CAD) and computer-aided manufacturing (CAM) methods have revolutionized the dental laboratory industry and led to breakthroughs in restoration production (2). Advantages of CAD/CAM systems include the production of higher and more uniform quality restorations by using commercially formed blocks of material, standardization of restoration shaping processes as well as reduced production cost and time. CAD/CAM systems also have some disadvantages. The scanning system has the limitation of finite resolution, which can result in edges that are slightly rounded. The point clouds obtained in scanning are transformed through a CAD software algorithm into a smooth and continuous surface, which can also lead to some internal inaccuracies further leading to interfering contacts at the incisal/occlusal edge and are proven to be detrimental if they occur at the margin.(3)

CAD/CAM technology can be divided into two categories according to the technique used: subtractive manufacturing technique and additive manufacturing technique. In the present study subtractive manufacturing technique has been used for the fabrication of zirconia crowns.

The marginal and internal fit is one of the key criteria for the clinical success of restorations (4). The deﬁnition of internal and marginal adaptation was described in 1989 by Holmes *et al.* (5) who stated that the internal gap is the perpendicular distance from the internal surface of the restoration to the axial wall of the preparation, whereas the same measurement at the margin is called the marginal gap. The vertical marginal misfit measured parallel to the path of draw of the casting is called the vertical marginal discrepancy. The horizontal marginal misfit measured perpendicular to the path of draw of the casting is called the horizontal marginal discrepancy. We choose the vertical marginal discrepancy for measuring the marginal gap because it is most critical due to cement solubility. Ideal marginal adaptation can produce less gingival irritation and cement dissolution. Excellent internal fit will facilitate crown seating without compromising retention and resistance forms. The large gap may result in plaque accumulation, marginal leakage, secondary caries and even crown failure. Theoretically, the internal space necessary for cement is 20 µm to 40 µm as reported by Fransson *et al.* (6). However, according to Martins LM *et al.* (7) study, the practical range for clinical acceptability of internal fit seems to be approximately between 50 to 100 µm. Beuer *et al.* (8) have reported that a 50 µm space in the chamfer area is expected to result in better seating at the margin area, but most authors agree to 120μm being the clinically acceptable maximum marginal gap for a good long-term prognosis, a value-based criterion established by Mc Lean and von Franhoufer (9).

Numerous *in vitro* studies have been conducted to evaluate the fit of CAD/CAM restorations and compare CAD/CAM with traditional casting protocols (10,11,12). However, a few studies have compared metal-ceramic crowns and CAD/CAM restorations in patients (13). Hence this study was designed to compare the marginal and internal fit of single unit monolithic zirconia crowns fabricated by CAD/CAM technique and metal-ceramic crowns fabricated by conventional technique. The null hypothesis was that no significant differences would be found in the marginal and internal fit of crowns fabricated with these two techniques.

## Material and Methods

A total of 10 participants needing a single restoration on endodontically restored teeth were selected from the outpatients of the Department of Prosthodontics and Crown & Bridge, JSS Dental College and Hospital, Mysuru for the study. The inclusion criteria were as follows: endodontically restored upper or lower molar with one or two cusps missing but ≥50% coronal tooth structure present. The exclusion criteria were as follows: a periodontal screening index >2, poor oral hygiene, bruxism and patients under the age of 18. An informed consent was obtained from all the participants enrolled in the study. The study protocol was approved by the Ethical Committee of JSS University.

For each participant two different types of crowns and a total 20 crowns were fabricated which were divided into two groups based on the manufacturing technique: Monolithic zirconia crowns fabricated by CAD/CAM technique (Lava ™,3M ESPE) (Group A) (n=10) and metal-ceramic crowns fabricated by conventional technique. (Group B) (n=10).

**Figure 1:**
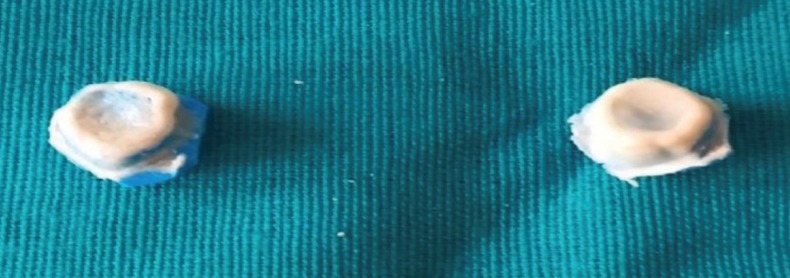
Silicone replicas of crowns.

Preparation of the restored teeth was performed with labially shoulder and lingually/palatally chamfer finish lines, where the location of the finish lines was considered optimal at an equigingival level. Guidelines for tooth preparation comprised of tapering of the axial walls by 6–10°, a circumferential reduction of the tooth between 1.2–1.5 mm, and an occlusal reduction of approximately 2 mm. All edges were rounded and smoothed out. Gingival retraction was done with retraction cords, sizes #0 and #1 (Ultrapak, Ultradent Products). The impressions of the prepared teeth for group A and B were made using polyvinyl siloxane material (Virtual; Ivoclar Vivadent) and reproduced in type IV (Kalrock Kalabhai Karson Pvt Ltd) dental stone to obtain the master casts. Temporary restorations were fabricated using a tooth coloured acrylic (DPI Self-Cure Tooth Moulding Powder) and cemented with non-eugenol temporary cement (Freegenol temporary Pack, GC Corporation, Tokyo, Japan) at the same appointment. The casts from group A were digitized using an optical 3D scanning instrument (Lava Scan S T;3M ESPE). All the copings were designed (Lava CAD; 3M ESPE) with the pre-set cement space at 40 µm and were then milled from pre-sintered monolithic zirconia blanks by a four-axis milling machine (Lava™ CNC 240; 3M ESPE). After the ﬁt check, all crowns were sintered to full density in a sintering furnace (Lava Furnace 200; 3M ESPE) for 8 hours. The deﬁnitive crowns were tried on their respective casts and glazed. The ﬁrst step in the preparation of metal-ceramic restorations was the wax-up of the copings onto the dies, which were previously varnished with two layers of die-spacer each of 20μm each (for standardization). Then, the wax pattern was attached to the crucible former with a sprue and placed into a casting ring coated with phosphate bonded investment, placed into the preheating furnace (WARMY 7 MANFREDI) with a heating rate of 2 to 5 °C/min, and heated to 900 to 950°C. Following this process, it was placed into the vacuum-pressure casting machine for induction heating (MANFREDI SAED Multihertz Ally Digital Induction Casting Machine System) and cast with a cobalt-based alloy (J BOND Ga, RUBY). After divesting, the castings were cleaned using airborne-particle abrasion with aluminium oxide powders (50μm), and the veneering ceramic (IPS Classic, Ivoclar Vivadent) was applied. A silicone replica technique was used to evaluate and compare the gap dimensions between the crowns and the prepared teeth, as described by Boening *et al.* (14) and Reich *et al.* (15) The inner surface of the crown was coated with light body polyvinyl siloxane (Virtual® Light Body Regular Set Wash Material, Ivoclar Vivadent) and positioned on the prepared tooth with ﬁnger pressure for 20s, and then fixed with a cotton roll while the patient closed his mouth. Excess silicone material was removed with a cotton pellet. After setting of the silicone layer (4:30 min), the crown was removed from the prepared tooth .The silicone material that adhered to the internal surface of the crown was stabilized with a regular set putty material of a different colour (Virtual® Putty Regular Set, Ivoclar Vivadent AG). After setting, both silicone materials were removed as one piece from each crown. The silicone replicas obtained are shown in Figure 1.

**Figure 2:**
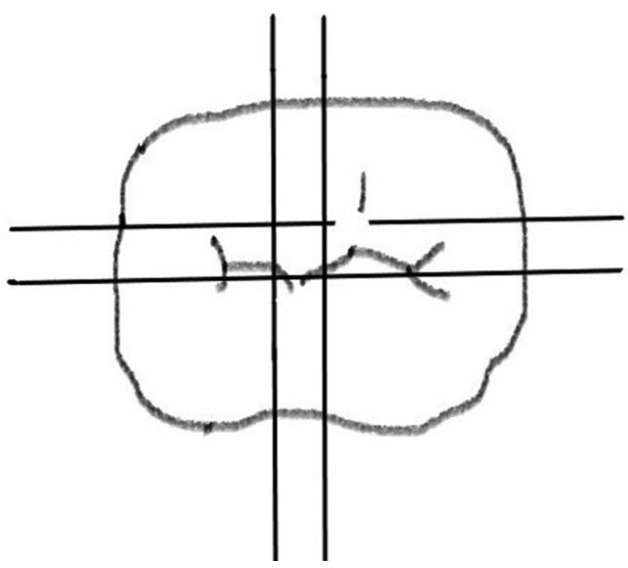
Schematic illustration of segmentation of silicone replica of crown (occlusal view).

**Figure 3:**
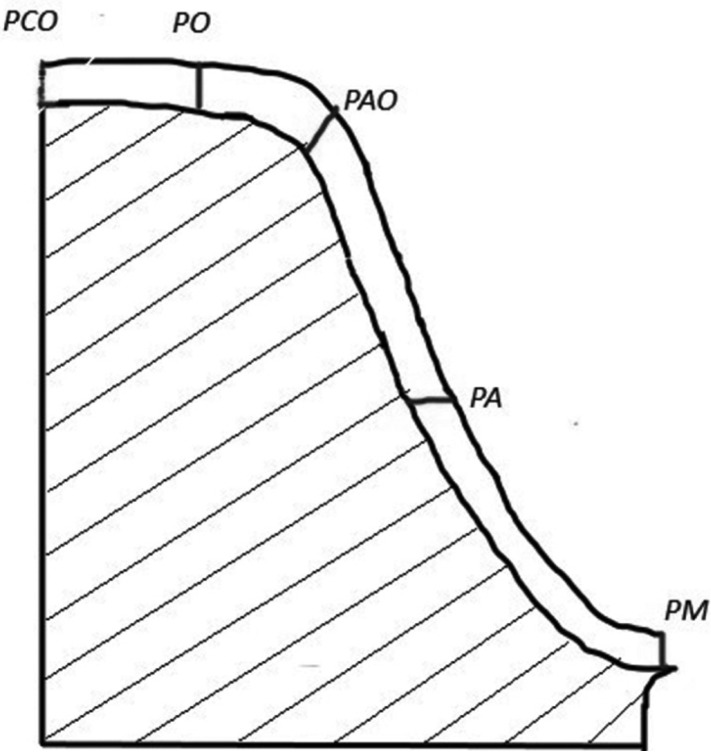
The predefined measuring points on each slice of the internal gap replica are: PM: marginal gap, PA: axial gap, PAO: axio-occlusal transitional gap, PO and PCO: occlusal gap.

The silicone replicas were cut with a sharp razor blade twice in the mesiodistal and twice in the buccolingual direction, resulting in four sections from each crown as shown in Figure 2. All sample measurements were carried out by one examiner. For each cross-section, five points have been measured in both mesiodistal and buccolingual direction. Hence, 10 points from each section and 40 points from each crown. For marginal gap measurement landmark (PM) has been used, for axial gap (PA), for axio-occlusal gap (PAO) and for occlusal gaps (PO, PCO) as shown in Figure 3. Point PM has been taken for marginal gap measurement and PA, PAO, PO, PCO for internal gap measurement. Internal gap was calculated by taking the means of values at points PA, PAO, PO and PCO. 40 points were measured for each crown in Group A and Group A consist of 10 CAD/CAM fabricated zirconia crowns. So, 400 points have been measured for Group A. Similarly, 40 points were measured for each crown in Group B and Group B consist of 10 metal-ceramic crowns. So, 400 points were measured for Group B and a total of 800 points have been measured. Cross-sections were adjusted horizontally on modelling wax to obtain a parallel orientation to the microscope’s plate and to achieve a rectangular observation angle. Replica film thickness was examined using a stereomicroscope (Lawrence and Mayo) at ×30 optical magnification and was digitally photographed using a digital camera in the Department of Oral Pathology, JSS Dental College and Hospital. A corresponding image manager (Aperio Image scope) was used for measurement. The statistical analysis was performed with SPSS 20.0 software (IBM Corp). Student’s paired t tests were used to compare marginal and internal gaps between both the groups. The cut off value for statistical signiﬁcance was set at α =0.05.

## Results

**Figure 4:**
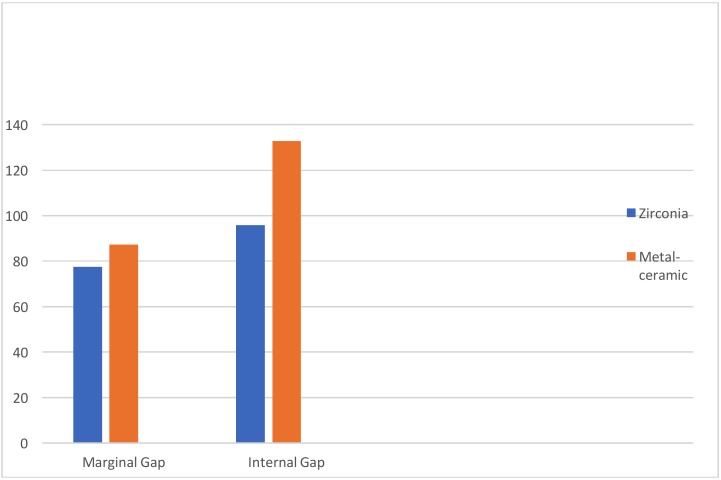
It shows that both marginal and internal gaps are more for metal-ceramic crowns (Group B) when compared to CAD/CAM fabricated zirconia crowns. (Group A).

The overall results for the mean, median, mode, SD for all landmarks for both the groups are shown in  Table 1. The mean marginal gap dimension at landmark PM was 77.4μm (SD 39.5μm) for Group A and 95.6 µm (SD 55.12μm) for Group B and. For landmark PA, the mean values were 57.1 (SD 29.5μm) for Group A and 80.23μm (SD 46.54μm) for Group B and. Landmark PAO had a mean of 104.71μm (SD 36.76μm) for Group A and 149.6 µm (SD 72.83μm) for Group B and. Point PO had mean values of 98.05μm (SD 43.82μm) and 155.14μm (SD 72.45μm) and for Group A and B, respectively. Landmark PCO showed a mean value of 89.11 µm (SD 23.97 µm) for Group A and 146.68 µm (SD 66.07) for Group B. Paired t test showed significant differences between the two groups for landmarks PM (*p*=.010), PA (*p*=.000), PAO (*p*=.000), PO (*p*=.010) and PCO (*p*=.000) ( Table 2) . Internal gap was calculated by taking average of landmarks PA, PAO, PO and PCO. Mean values for internal gap was 87.24 (SD 21.7 µm) for zirconia and 132.91 µm (SD 50.63 µm) for metal-ceramic as shown in Figure 4.

**Table 1:**
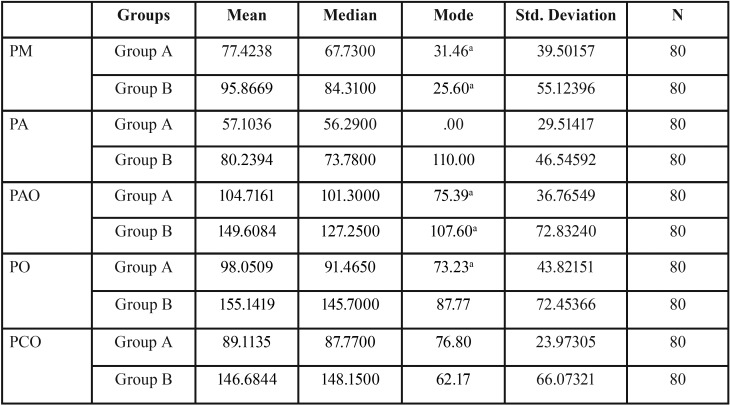
Mean values (standard deviation) for the gap thickness at different evaluated regions (values in µm) for the experimental groups. (n=80 where n stands for the number of points measured for each pre-determined site).

**Table 2:**
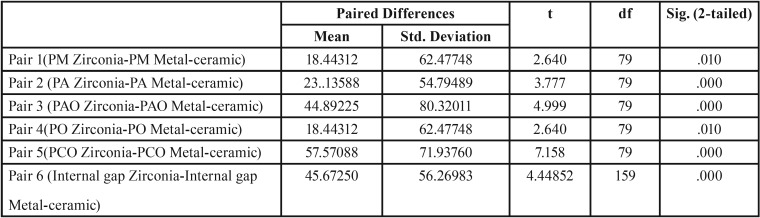
The table shows that significant differences were present between both Zirconia and metal-ceramic crowns at all the landmarks measured.

## Discussion

This study evaluated the marginal and internal adaptation of CAD/CAM fabricated zirconia monolithic crowns and compared them to conventional metal-ceramic crowns. The results supported rejection of the null hypothesis as significant differences were found in the marginal and internal fit between the two crowns.

Previously many studies have been done to evaluate the marginal and internal fit of crowns by using various methods and materials. The major drawbacks of comparing the results of different studies include the absence of a standardized methodology and the fact that many factors can inﬂuence the results of the study. One of the factors is the different measurement methodologies used. Although various protocols have been proposed to analyse marginal precision, no guidelines exist regarding how to perform gap measurements; therefore, variability exists in the results obtained from different techniques used to record the data (12,13).

There are several basic methods used to measure marginal and internal gaps; direct view (external microscopic examination), cross-sectional technique after cementation and embedding (internal microscopic examination), impression technique (internal replica approach), weighing the light-body additional silicone, and explorer and visual examination .In the present study, impression technique (replica method) was used for evaluating marginal and internal fit, which is considered as a reliable and non-invasive method for measuring marginal and internal fit. In this technique, the cement is replaced with an impression material, and the restoration is placed on the abutment. The restoration and impression material are separated from the abutment, and the thickness of the cement layer analogue is measured. However, the impression replica technique has certain limitations such as difficulty in identifying the crown margins and finish lines, tearing of the elastomeric film upon removal from the crown and mistakes in sectioning plane which will eventually lead to overestimated measurements (16).

Another factor that affects the fit of restoration is cementation. Borges *et al.* (17) have stated that cementation increases the marginal gap whereas as Gonzalo *et al*. (18) did not find any significant difference in vertical marginal discrepancies of crowns before and after cementation. In the present study measurements were performed on crowns seated with light body silicone which are comparable to the castings cemented with zinc phosphate cement (19).

The studies have shown significant differences in vertical marginal discrepancies after porcelain veneering (20). In the present study replicas of monolithic zirconia crowns were made. Hence further studies are required to compare zirconia crowns veneered with porcelain and metal-ceramic crowns.

The location of measurement site is another factor influencing the result of the studies which has to be standardized for better credibility of the study.

The skill of the dental technician also has an important role in producing well adapted restorations. Hence in the present study all the crowns are fabricated by the single investigator (21).

Another factor affecting the results of the study is milling axis used in CAD/CAM. Most cutting tools are incapable of cutting sharp internal angles which results in an increased marginal gap. To avoid this problem, a spacer parameter has to be chosen in the CAD/CAM system, or the fit of the crown has to be corrected by the technician, using a handpiece, during the laboratory fitting procedure. Beuer *et al.* (8) reported that the provision for internal relief in the CAD/CAM process called ‘radius cutter’ adds about 50 um space at the chamfer area, is expected to result in better seating at the margin. In the present study, cement space for all copings were 40 µm according to manufacturer instructions.

The current study had clinically accepTable results of 95.6 µm for marginal gap and 132.91 µm for internal gap in the metal-ceramic group and 77.4 µm of marginal gap and 87.24 µm of internal gap in the CAD CAM zirconia group. Though a better fit was obtained with zirconia crowns, 7 out of 10 patients opted for metal-ceramic crowns because of their better strength properties1, after mechanical properties of both the crowns were explained to the patient. It shows that metal-ceramic crowns remain one of the most popular choice for patients. The marginal gap obtained for monolithic zirconia crowns (77.4 µm) was similar to the results obtained by Reich *et al.* (15) (80 µm) whereas the values obtained for occlusal and axio-occlusal gaps were lower than that of Reich *et al.* Freire *et al.* (21) conducted a similar study but they have used stainless steel master dies and SEM analysis for their study and obtained a marginal gap of 58.05 µm for monolithic zirconia crowns and 57.42 µm for metal ceramics, a finding lesser than the values obtained in the present study. Tamac *et al.* (13) performed a similar study on metal-ceramic crowns, they used the same techniques and obtained a marginal gap of 86.64 µm for CAD/CAM and 75.92 µm for traditional casting technique. The mean value obtained for internal gap was 87.24 µm in CAD/CAM zirconia group and similar findings have been obtained by Nakamura *et al.* (22), Lee *et al.* (23), and Beuer *et al.* (24).

Most of the investigators used a single master model and subsequently duplicated the working models using impressions (10,11,18). The shortcoming of this technique is the impact of impression making on the reproduction of working models. The present study was a clinical evaluation, and the results are much closer to reality than an *in vitro* study; however, there were certain limitations which are as follows: 1) In the present study 40 reference points were measured per specimen which can be increased for better credibility of the study 2) during the clinical try-in, only ﬁnger pressure was used, which might result in different thicknesses of the silicone layers 3) only conventional system and CAD/CAM grinding systems were evaluated but more milling systems should be studied in the future to compare the ﬁt of crowns 4) The inﬂuence of the abutment tooth type on the ﬁt of a restoration is controversial. In this study preparation was done only for molar crowns but more studies are required with comparison between different abutment tooth for marginal and internal fit.

Hence in future long-term studies are required with more sample size and a special device has to be designed to allow uniform loading of the crown while the silicone layer will be setting.

## Conclusions

Within the limitations of this study the following conclusions can be drawn:

1. Marginal discrepancy, occlusal discrepancy and axial discrepancy of both systems are clinically acceptable.

2. The CAD/CAM crowns demonstrated a better accuracy of fit when compared to metal-ceramic crowns fabricated by conventional technology.

3. The mean marginal and internal gaps varied signiﬁcantly within a measured tooth. The occlusal gap was more compared to marginal and axial gap for both the crowns.
